# Dietary habits and the gut microbiota in military Veterans: results from the United States-Veteran Microbiome Project (US-VMP)

**DOI:** 10.1017/gmb.2021.1

**Published:** 2021-04-28

**Authors:** Diana P. Brostow, Christopher E. Stamper, Maggie A. Stanislawski, Kelly A. Stearns-Yoder, Alexandra Schneider, Teodor T. Postolache, Jeri E. Forster, Andrew J. Hoisington, Christopher A. Lowry, Lisa A. Brenner

**Affiliations:** 1 VA Rocky Mountain Mental Illness Research Education and Clinical Center (MIRECC), Rocky Mountain Regional VA Medical Center (RMRVAMC), Aurora, CO, USA; 2 Department of Physical Medicine & Rehabilitation, University of Colorado Anschutz Medical Campus, Aurora, CO, USA; 3 Department of Psychiatry, University of Colorado Anschutz Medical Campus, Aurora, CO, USA; 4 Military and Veteran Microbiome: Consortium for Research and Education, Aurora, CO, USA; 5 Mood and Anxiety Program, University of Maryland School of Medicine, Baltimore, MD, USA; 6 Department of Veterans Affairs, VISN 5 MIRECC, Baltimore, MD, USA; 7 Department of Systems Engineering & Management, Air Force Institute of Technology, Wright-Patterson AFB, OH, USA; 8 Department of Integrative Physiology, University of Colorado Boulder, Boulder, CO, USA; 9 Center for Neuroscience, University of Colorado Boulder, Boulder, CO, USA; 10 Center for Neuroscience, University of Colorado Anschutz Medical Campus, Aurora, CO, USA; 11 Department of Neurology, University of Colorado Anschutz Medical Campus, Aurora, CO, USA

**Keywords:** Diet, dietary quality, gut microbiota, microbiome, Veterans, Western diet

## Abstract

Dietary patterns influence gut microbiota composition. To date, there has not been an assessment of diet and gut microbiota in Veterans, who have a history of unique environmental exposures, including military deployment, that may influence associations between diet and gut microbiota. Our aim was to characterise Veteran habitual dietary intake and quality, and to evaluate correlations between diet and gut microbiota. We administered Food Frequency Questionnaires (FFQs) and collected stool samples from 330 Veterans. FFQ data were used to generate Healthy Eating Indices (HEI) of dietary quality. Exploratory factor analysis was used to identify two dietary patterns we defined as “Western” and “Prudent.” Stool samples underwent 16S rRNA gene sequencing, and the resulting data were used to evaluate associations with dietary variables/indices. Analyses included linear regression of α-diversity, constrained analysis of principal coordinates of β-diversity, and multivariate association with linear models and Analysis of Composition of Microbiomes analyses of dietary factors and phylum- and genus-level taxa. There were no significant associations between dietary patterns or factors and α- or β-diversity. At the phylum level, increasing HEI scores were inversely associated with relative abundance of Actinobacteria, and added sugar was inversely associated with abundance of Verrucomicrobia. Veterans largely consumed a Western-style diet, characterised by poor adherence to nutritional guidelines.

## Introduction

The gut microbiota plays a significant role in host functioning, and a growing body of evidence explores its impact on metabolic pathology, immune regulation (Mazidi et al., 2016; Thaiss et al., 2016) and neurological and cognitive functioning (Dinan and Cryan, 2017). As such, the gut microbiota is increasingly viewed as a potential target for therapeutic intervention (Belizário and Napolitano, 2015). A wide array of genetic factors and environmental exposures influence the microorganisms that comprise the gut microbiota, and, among environmental exposures, diet has been proposed as a potentially significant modulator (Graf et al., 2015).

Short-term dietary interventions can alter gut microbiota composition (Faith et al., 2013), and dietary intake in the days preceding stool sampling can exert a significant influence on species diversity (Johnson et al., 2019). Nevertheless, there is increasing evidence that lifelong dietary habits exert a more significant and lasting influence on microbial composition, as observed in a recent study by Partula et al. (2019). There is also evidence to suggest that regular consumption of a greater variety of nutritious foods, as measured for example by the Healthy Eating Index (HEI; Krebs-Smith et al., 2018), is associated with a more diverse gut microbiota (Asnicar et al., 2021; Claesson et al., 2012; Menni et al., 2021; Vujkovic-Cvijin et al., 2020). Moreover, broader eating patterns (eg. “Prudent”-style and “Western”-style) may also be useful measures of nutritional influences on microbial composition. While there is a growing body of research in these areas, many different populations have yet to be examined. U.S. military Veterans are particularly underexamined in both microbiota and nutrition research. The majority of nutrition studies in U.S. military Veterans have focussed on specific disease etiology rather than on overall dietary habits (Collins et al., 2020), though a single systematic review and meta-analysis revealed the health benefits of a Mediterranean or “Prudent-style” diet in U.S. Veterans (Bloomfield et al., 2016). Although the gut microbiome is a potential mediator of these effects, little is known about the relationship between dietary patterns and the gut microbiome diversity or community composition in this population. U.S. Veterans currently number nearly 19 million (Veteran Population – National Center for Veterans Analysis and Statistics, 2016) and they are more likely than the general population to have unique microbial exposures, such as living in foreign environments (military deployment), and experiencing higher rates of homelessness (Brenner et al., 2018). Veterans comprise a unique population; therefore, the primary aim of the present work was to evaluate the associations between dietary characteristics and gut microbiota composition in a sample of U.S. military Veterans.

## Methods

### Study population

The United States-Veteran Microbiome Project (US-VMP) has previously been described (Brenner et al., 2018). Briefly, U.S. Veterans are eligible to participate, and data collection has continued since May 2016. For this study, data from 330 U.S. Veterans were drawn from an in-person baseline assessment. At that visit, Veterans completed clinical interviews and self-report measures related to demographics and health histories, as well as provided skin, oral and stool microbiome samples.

### Ethics

This study was conducted according to the guidelines laid down in the Declaration of Helsinki and all procedures involving human participants were approved by the Colorado Multiple Institutional Review Board (COMIRB). Written informed consent was obtained before Veterans participated in any study procedures.

### Data collection

#### Dietary data

Dietary data were evaluated using the Harvard Willett Food Frequency Questionnaire (FFQ; Willett et al., 1985) that was administered by trained research assistants. The FFQ consists of over 100 items in 18 categories, including: nutritional supplements; dairy foods; dairy substitutes; fruits; non-starchy vegetables; animal proteins (eggs, meat, fish and seafood); carbohydrate-rich foods (grains, cereals, breads, pasta and starchy vegetables); soft drinks (sugar-sweetened and artificially-sweetened); other non-alcoholic beverages (coffee, tea and fruit juice); alcoholic beverages; desserts and baked goods; condiments (eg. ketchup and salad dressing) and types of cooking fats and oils, consumed in and outside the home. Participants were asked to report how often they had consumed a standardised portion of each type of food/beverage in the previous year (eg. half a cup of cooked beans, one orange and five-ounce glass of wine), with possible responses ranging from “never” to “6 or more times per day.”

#### Covariates

Standard demographic questions and information regarding military history were also collected. Other demographic data including smoking history, as well as the Charlson Comorbidity Index (VIReC, 2014, Rev. September 2017) were obtained from the electronic medical record via the Veterans Affairs Corporate Data Warehouse.

#### Gut microbiota

The data collection for the US-VMP has been previously described in detail (Brenner et al., 2018), including microbiota sample collection procedures (see **Supplementary Material)**. Sample DNA was extracted from fecal samples using the PowerSoil DNA extraction kit (Cat. No. 12955-4, Qiagen, Valencia, CA). The 16S rRNA gene sequences in isolated DNA were polymerase chain reaction (PCR)-amplified using GoTaq Hot Start Master Mix (Cat. No. M5133, Promega, Madison, WI). PCR products were cleaned and normalised using the SequalPrep Normalisation Kit (Cat. No. A1051001, ThermoFisher, Waltham, MA) following manufacturer’s instructions. The normalised amplicon pool was sequenced on an Illumina MiSeq using V3 chemistry and 2 × 300 sequencing run. All library preparation and sequencing were conducted at the University of Colorado Boulder BioFrontiers Next-Gen Sequencing core facility.

Sequencing data were processed using the Quantitative Insights into Microbial Ecology programme (QIIME2 v. 2019.10; Bolyen et al., 2019). The Deblur (Amir et al., 2017b) algorithm was used to denoise demultiplexed sequences. SEPP (Janssen et al., 2018) analysis was performed to remove sequences that were not 75 per cent similar to any record in the tree. Quality-filtered sequences were assigned taxonomic classification based on the silva_12.8 database (Quast et al., 2012). Samples that were shipped to the research facility and had taxa that are known to “bloom” during shipping were removed as previously described by Amir et al. (2017a). Additionally, taxa identified by Bokulich et al. (2019), to “bloom” with increased time spent at room temperature were also considered for removal. Details regarding the “deblooming” analysis can be found in Supplementary Material. For α- and β-diversity and taxonomic evaluations, samples were rarefied to a level of 2,535 sequences per sample.

### Statistical analyses

As a descriptive and exploratory cross-sectional study, feasibility of recruitment determined the final sample size (*N* = 330).

FFQ responses were converted and standardised as daily intakes using Harvard FFQ guidelines (Willett et al., 1985). For example, a response of “2–4 times per week” was converted to 0.43 servings per day. For specific nutrients or ingredients (eg. sodium, caffeine and artificial sweeteners), portion sizes were multiplied by frequencies of intake, and the subsequent intakes were summed across all foods using food composition data from the U.S. Department of Agriculture (Gebhardt et al., 2008).

We applied the HEI (2015 version; Krebs-Smith et al., 2018) to evaluate dietary quality. The HEI-2015 was created to assess adherence to the 2015–2020 U.S. Dietary Guidelines for Americans established by the U.S. Departments of Agriculture, and Health and Human Services (US Department of Agriculture, 2015), and can be used to identify 13 different energy-adjusted variables. These include items that assess frequencies of intake of food groups (eg. whole grains, saturated fat and added sugars), and proportional intakes (eg. density of total protein or sodium per 1,000 kilocalories). HEI analysis yields individual subscales for each variable, and as an aggregate score ranging from 0 to 100, with 0 being no adherence to dietary guidelines, and 100 being perfect adherence.

**Figure 1:**
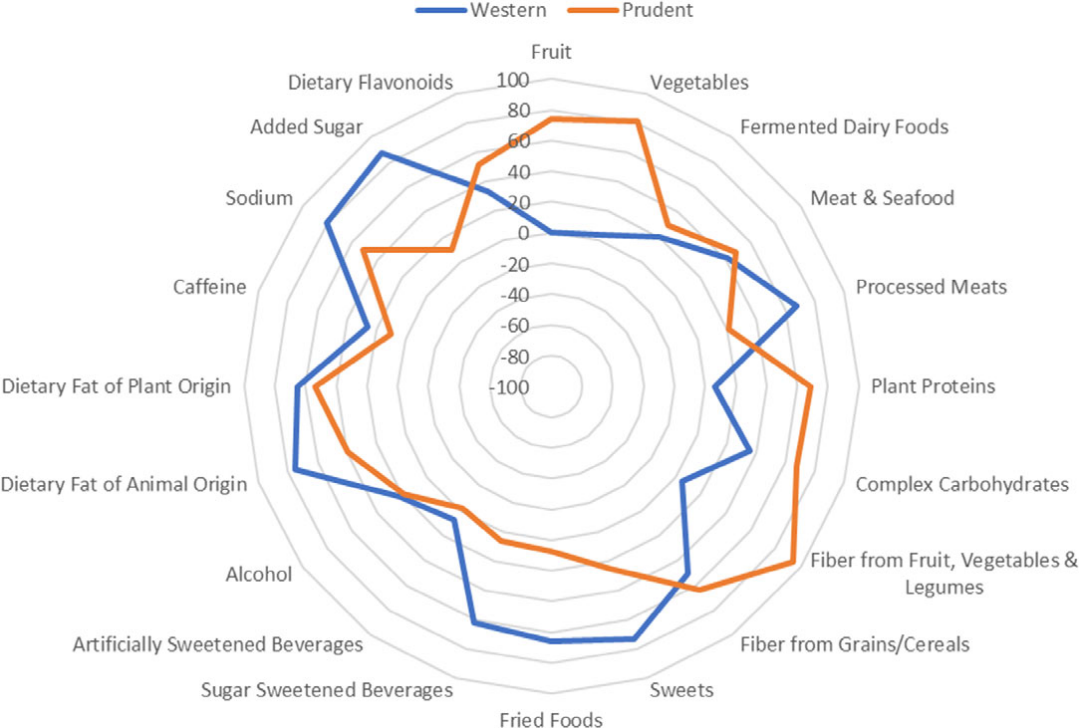
Radar graph of factor loadings that characterise each dietary pattern. The blue line represents the distribution of factor loadings for the Western dietary pattern, the orange line for the Prudent dietary pattern.

To evaluate dietary patterns, items from the FFQ were first classified into 20 groups based on nutritional characteristics (eg. plant proteins and dietary fat of animal origin; see Supplementary Table S2). Other groups included foods or nutrients of potential interest that have previously been examined in gut microbiota research (eg. lacto-fermented dairy products and dietary flavonoids) (Singh et al., 2017). Before applying exploratory factor analysis (principle component analysis) to the food groups, the data were tested and found to be suitable for factor analysis using the Kaiser–Mayer–Olkin test (*Measure of Sampling Adequacy* = 0.805), and the Bartlett test of Sphericity (*p* < 0.001). We used varimax rotation to obtain combinations of correlated factors, and using an eigenvalue criterion of 1.0, identified two distinct factors (dietary patterns) that we labeled “Western” and “Prudent.” A scree plot (not pictured) confirmed the two-factor solution, with the Western and Prudent dietary patterns explaining 58 and 17 per cent of the variance, respectively. In Figure 1, a radar graph of factor loadings for each dietary pattern is presented. The Western dietary pattern was characterised primarily by more frequent consumption of processed meats (eg. sausages, bacon and cold cuts), added sugar, sodium and dietary fat of animal origin. More frequent intakes of vegetables, plant proteins, fiber from fruit/vegetable/legume sources, and dietary fat of plant origin were characteristic of the Prudent dietary pattern. Items with factor loadings <0.4 for both the Western and Prudent dietary patterns included fermented dairy products, artificially sweetened beverages, alcohol and caffeine. The factor scores (ie. Prudent and Western scores) generated for each participant were then split at the median to represent high or low adherence to each pattern. The combination of each participants’ categorised pattern scores was then used to create four final categories representing Very Low, Low, Moderate and High adherence to a Western-style diet. Specifically, participants with Western pattern scores less than the median and Prudent scores greater than or equal to the median were placed in the final category of Very Low adherence to the Western-style diet; those with low scores for both patterns were placed in the final category of Low adherence; those with high scores for both patterns were placed in the final category of Moderate adherence; and, those with low Prudent scores and high Western scores were placed in the final category of High adherence to the Western-style diet. These final four categories were used for subsequent microbiota analyses.

Based on previous literature, we selected 22 dietary variables (see Figure 4 for the complete list) for microbiota diversity analyses. For α-diversity, the metrics assessed were: (1) observed Operational Taxonomic Units (OTUs); (2) Shannon diversity and (3) Faith’s phylogenetic diversity (PD). Linear regression models were conducted for each α-diversity metric as a function of each dietary variable. For β-diversity, in order to determine the strongest correlates of the gut microbiota composition, we used the model building tool for constrained ordination methods *envfit* from the *vegan* package in R based on adjusted *R^2^* value using the weighted and unweighted UniFrac distance metrics (Oksanen et al., 2018). α-diversity regression analyses and β-diversity model building were conducted while controlling for total energy intake, gender, race (Caucasian/White; Black; other), number of military deployments, current homeless status, marital status (married/partnered or single), education (high school diploma equivalent or less; some college, no degree; post-secondary degree, post-graduate degree), and lifetime smoking status. In addition, we used the Benjamini–Hochberg false discovery rate (FDR) (Benjamini and Hochberg, 1995) to correct for the 22 dietary variables examined with a significance cutoff of FDR*p* < 0.10. Multivariate association with linear models (MaAsLin) was used in the Galaxy platform v.1.0.1 (Afgan et al., 2018) to examine the association between dietary variables and taxa at the phylum and genus levels with multivariate adjustment for all other dietary covariates, and correction for multiple comparisons made for the number of taxa examined, again using FDR (Benjamini and Hochberg, 1995) with a significance cutoff FDR*p* < 0.10. We used Analysis of Composition of Microbiomes(Mandal et al., 2015) to determine differentially abundant taxa by quartile of adherence to a Western dietary pattern. All statistical analyses were performed with QIIME2 v. 2019.10, the open source statistical package R v.3.5.1, or SAS v.9.4.

## Results

### Cohort characteristics

A total of 330 Veterans completed all microbiota and dietary measures. Demographic and medical characteristics are presented in Supplementary Table S3. The majority of the cohort was male, Caucasian/White, single or divorced/separated, and had completed at least some post-secondary education. Nearly 9 per cent were homeless at the time of data collection, and a further 42 per cent reported having previously experienced homelessness at least once in their lives. Over three-quarters of the cohort were current or former tobacco users.

**Figure 2:**
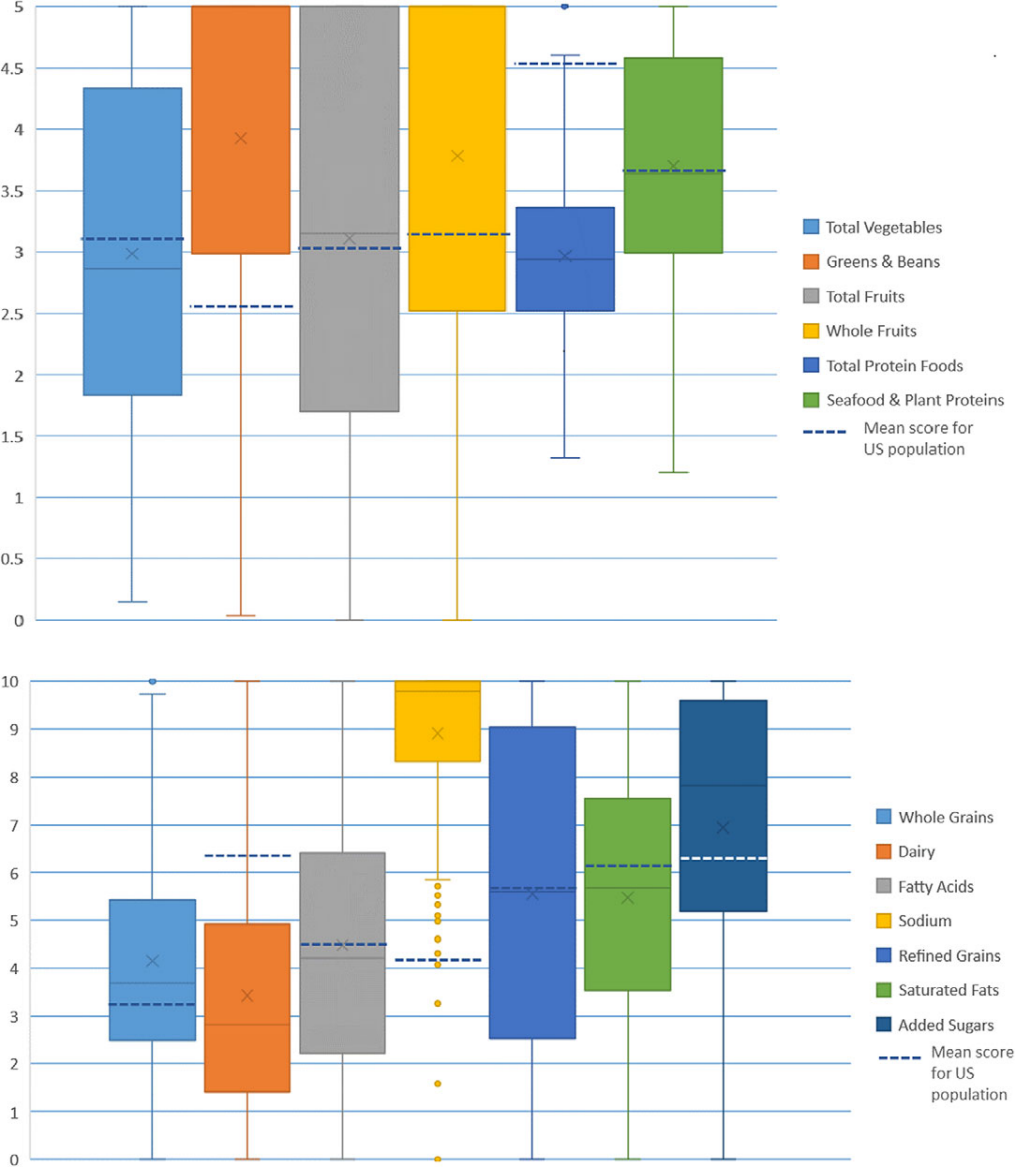
(a and b) Box-and-whisker plots of Healthy Eating Index (HEI) component scores for the United States-Veteran Microbiome Project (US-VMP) cohort, with comparisons to mean scores for the general U.S. population.

The mean and median total HEI scores were both 59, which is also the U.S. mean score (Center for Disease Control and Prevention*,* 2016). HEI component sub-scores for food groups are presented in Figure 2,b. Overall, Veterans largely aligned with the general population in their scores for total vegetables, total and whole fruit, seafood and plant proteins, dietary fat (fatty acids) and refined grains. Compared to the general population, however, Veterans scored higher for green vegetables and legumes (Greens and Beans), and most notably, added sugars and sodium. For HEI scores for proportions of energy intake (not pictured), many of the data distributions were skewed. As a proportion of 1,000 calories, 75 per cent or more of Veterans scored on the lowest end of total vegetables, greens and beans, whole fruit and dairy.

Mean intake frequencies for selected food groups thought to exert a significant influence on microbial composition (Claesson et al., 2012) are presented in Supplementary Figure S2. Veterans infrequently consumed fermented dairy products (eg. Veterans reported rarely, if ever, consuming yogurt in particular), and the majority reported that they “never” consumed artificially sweetened beverages. Closer examination of legume intake showed that Veterans also infrequently consumed any varieties of beans, with only 11 per cent reporting that they consumed beans once per week or more, indicating that their scores in the greens and beans HEI component was largely driven by consumption of green vegetables. Fewer than 20 per cent of respondents reported consuming one or more alcoholic beverages on a daily basis. Among other dietary characteristics, the majority of the cohort (68 per cent) reported having taken a nutritional supplement in the past year, particularly a psyllium fiber supplement (69 per cent), though with uncertain frequency.

### Dietary variables and α-diversity indices

**Figure 3:**
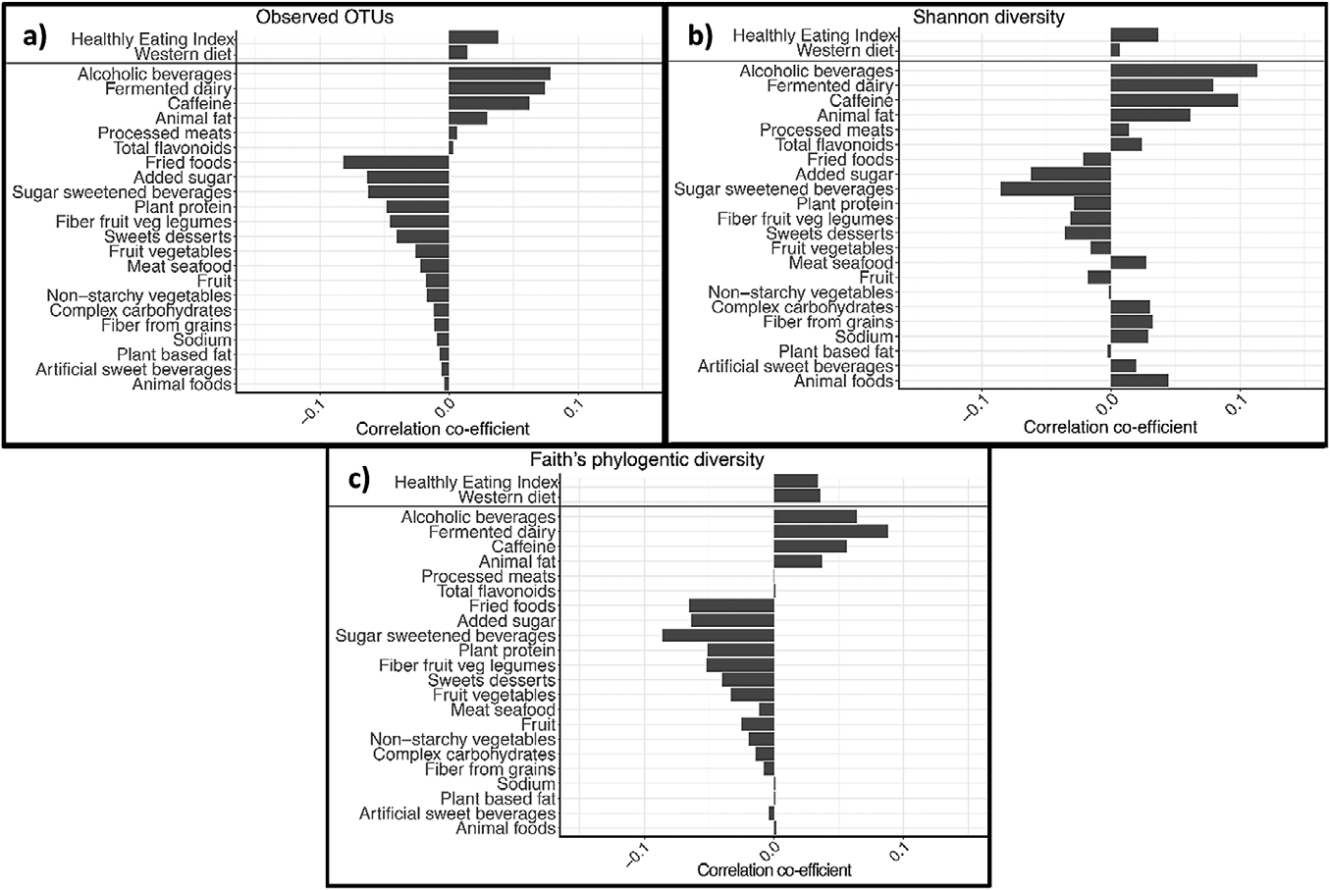
(a–c) Demographic-, comorbidity- and energy-adjusted correlation coefficients between dietary variables and α-diversity indices (Observed OTUs, Shannon diversity, and Faith’s phylogenetic diversity) in the United States-Veteran Microbiome Project (US-VMP) cohort, *N* = 330.

Figure 3,c presents the correlation coefficients between dietary variables and three α-diversity indices. Corresponding regression estimates are presented in Supplementary Table S4. Caffeine and animal fat showed significant direct associations with Shannon indices of α-diversity (*rho* = 0.06, *p* = 0.01 and *rho* = 0.05, *p* = 0.03, respectively), whereas sugar sweetened beverages showed a significant inverse association with the Shannon index of α-diversity, *rho* = −0.05, *p* = 0.05. Added sugars showed a significant inverse association across all three metrics of α-diversity (Observed OTUs *rho* = −0.07, *p* = 0.02, Shannon diversity *rho* = −0.06, *p* = 0.01, and Faith’s PD *rho* = −0.06, *p* = 0.02). After adjustment for multiple comparisons, however, no single dietary variable was significantly associated with any index of α-diversity.

### Dietary variables and ß-diversity indices

**Figure 4:**
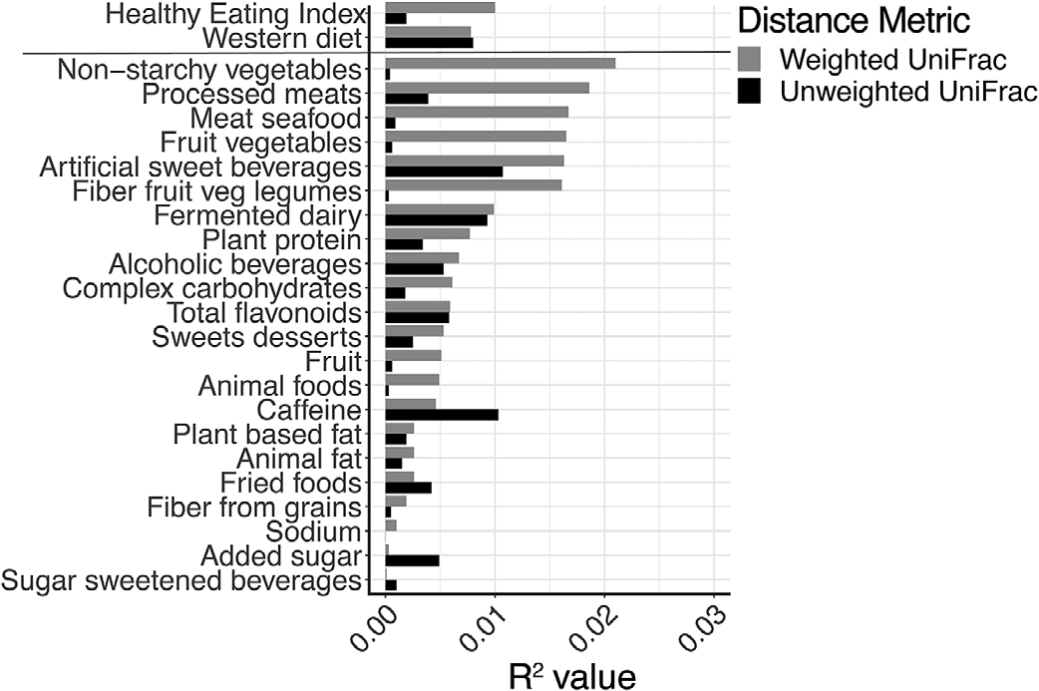
Proportions of explained variation in gut microbiome composition explained by dietary variables (Weighted and Unweighted Unifrac dissimilarities), adjusted for demographic variables, medical comorbidities, total energy intake, and military deployment history.

Associations between dietary variables and β-diversity are presented in Figure 4, with corresponding *R^2^* estimates presented in Supplementary Table S5. After adjustment for multiple comparisons, no dietary variables were associated with dissimilarity in microbial composition among participants.

### Dietary variables and relative abundances of phyla and genera

Phylum level microbiota composition varied among participants (see Supplementary Figure S3). The six most abundant phyla, in descending order, were Firmicutes, Bacteroidetes, Actinobacteria, Proteobacteria, Verrucomicrobia and Tenericutes. Table 1 presents associations between dietary variables and taxa. To limit the number of comparisons, we analysed food groups from the FFQ thought to be particularly associated with gut microbial composition: animal foods; fruit and vegetables; plant proteins and added sugars, as well as Total HEI scores. Added sugar intake was inversely associated at the phylum level with Verrucomicrobia (*rho* = –0.0003; FDR corrected-*p* = 0.03). Also at the phylum level, increasing total HEI scores were inversely associated with Actinobacteria (*rho* = –0.002; FDR corrected-*p* = 0.09). We did not observe any significant associations between these variables and microbiota at the genus level (Table 1). Lastly, there were no significant differences in relative abundances in taxa by quartile of adherence to a Western-style dietary pattern (data not shown, *p* > 0.05).

**Table 1.** Associations between selected dietary variables and relative abundances of taxa.^a^

Dietary Variable^b^	Phylum	MaAsLin coefficient	% Coverage^c^	*P-*value	FDR*p*-value^d^
Animal foods	Firmicutes	–0.00584	100	0.211	0.449
	Bacteroidetes	0.00500	100	0.418	0.449
	Proteobacteria	0.00196	96.4	0.380	0.449
	Verrucomicrobia	–0.00118	45.8	0.449	0.449
Fruit and vegetables	Bacteroidetes	0.00565	100	0.480	0.765
	Firmicutes	–0.00212	100	0.682	0.765
	Actinobacteria	–0.00402	99.1	0.320	0.765
	Proteobacteria	0.00086	96.4	0.765	0.765
Plant protein	Bacteroidetes	0.00957	100	0.641	0.847
	Firmicutes	0.00530	100	0.726	0.847
	Tenericutes	0.00297	19.4	0.416	0.774
	Synergistetes	0.00019	9.09	0.418	0.862
Added sugar	Firmicutes	0.00016	100	0.538	0.731
	Bacteroidetes	–0.00018	100	0.640	0.731
	Proteobacteria	–0.00023	96.4	0.107	0.286
	Verrucomicrobia	–0.00027	45.8	0.004	**0.033**
	Bacteroidetes	0.00019	100	0.899	0.898
Healthy Eating Index	Actinobacteria	–0.00169	99.1	0.024	**0.085**
	Proteobacteria	0.00050	96.4	0.356	0.623
	Fusobacteria	0.00001	20.9	0.859	0.899
	Firmicutes	–0.00584	100	0.211	0.449
Dietary Variable^b^	Genus	MaAsLin coefficient	% Coverage^c^	*p-*value	FDR*p*-value^d^
Animal foods	*Blautia*	–0.00089	98.5	0.668	0.936
	*Ruminococcus*	0.00033	93.6	0.773	0.936
	*Faecalibacterium*	–0.00077	93.3	0.812	0.936
	*Alistipes*	–0.00005	90.3	0.984	0.984
Fruit and vegetables	*Bacteroides*	0.01655	99.4	**0.020**	0.666
	*Blautia*	0.00225	98.5	0.403	0.849
	*Faecalibacterium*	0.00342	93.3	0.410	0.849
	*Parabacteroides*	0.00099	92.7	0.767	0.929
Plant protein	*Bacteroides*	0.02238	99.4	0.223	0.747
	*Blautia*	0.01097	98.5	0.112	0.665
	*Ruminococcus*	0.00027	93.6	0.939	0.973
	*Faecalibacterium*	–0.00137	93.3	0.897	0.973
Added sugar	*Bacteroides*	–0.00076	99.4	0.034	0.676
	*Blautia*	7.61E-05	98.5	0.564	0.958
	*Ruminococcus*	–1.22E-06	93.6	0.853	0.967
	*Faecalibacterium*	–0.00016	93.3	0.434	0.958
Healthy Eating Index	*Bacteroides*	–0.00166	99.4	0.231	0.761
	*Blautia*	–0.00076	98.5	0.140	0.726
	*Ruminococcus*	0.00012	93.6	0.592	0.875
	*Faecalibacterium*	0.00018	93.3	0.817	0.937

a Derived from participants’ responses to the food frequency questionnaire (FFQ). Bolded p-values indicate a value </ = 0.05.

b Most abundant relative taxa after multiple comparison testing using multivariate association with linear models (MaAsLins) in the United States-Veteran Microbiome Project (US-VMP) cohort, *N* = 330.

c Percentage of samples with specified bacterial taxa in the US-VMP cohort.

d *p* value after multivariate adjustment for all other dietary variables, and correction for multiple comparison testing using a false discovery rate (FDR) of 0.10.

## Discussion

In this study, we examined habitual dietary intake and quality, as well as associations between diet and various metrics of gut microbial diversity and community structure in a sample of 330 U.S. military Veterans. Veterans’ dietary quality, as measured by the HEI, matched estimates of national average diet quality. Factor analysis confirmed that there were two general dietary patterns among Veterans, which were distinctly characterisable as Western-style and Prudent-style eating habits. We did not observe significant associations between dietary variables and α- or β-diversity metrics, though we did find significant correlations between added sugar and total HEI with the relative abundance of specific phyla.

Phyla-level correlations are relatively broad and are less robust indicators of diet-microbiome associations than more specific microbial signatures. Nevertheless, these findings highlight the possible phyla that merit further investigation. In our study, Actinobacteria was inversely associated with HEI scores. Previous studies have reported an inverse association between Actinobacteria and increased intakes of dietary fiber (Wu et al., 2011), and a positive association with high-fat, high-animal food intake in human populations (Rinninella et al., 2019). Recently, Partula et al.’s (2019) study of a large community-based cohort observed a direct association between consumption of high-fat sweets and increased relative abundance of Actinobacteria. In addition, we found that added sugar was significantly associated with decreased relative abundance of Verrucomicrobia. A small study (Egshatyan et al., 2016) observed a decreased relative abundance of Verrucomicrobia in participants with diabetes-related impaired glucose tolerance, suggesting there may be a correlation with dietary sugar; however, research in this area is still scant.

A variety of factors may explain the overall lack of distinct associations between dietary variables and gut microbial composition observed in this cohort. In this study, we used the HEI to assess dietary quality. While our cohort’s dietary habits were similar to those of the general U.S. population, there was evidence to suggest that Veterans consume poorer-quality foods more frequently, specifically foods high in added sugar and sodium. While the general population consistently exceeds maximum recommended intakes of added sugar and sodium (24–36 g/day and 2,300 mg/day, respectively; US Department of Agriculture, 2015), our data suggest that Veterans on average exceed those recommendations even further. Skewed data for several proportional intake scores also suggest that many Veterans consumed a largely uniform Western-style diet. Despite the presence of two distinct dietary patterns, most Veterans derived relatively little of their caloric energy from fiber-rich or probiotic-containing foods, which may have made associations with microbial composition more difficult to detect. These findings may be random artifacts of data derived from a single, cross-sectional FFQ, the use of which is a key limitation of our study. Of note, previous studies of other populations have observed significant correlations between decreased microbial diversity and a Western-style dietary pattern (Davis et al., 2017; Graf et al., 2015; Partula et al., 2019). All the same, given the relative paucity of data on Veterans’ general dietary habits, our findings comprise an important step towards rectifying these gaps in knowledge.

Secondly, parsing the individual contributions of various factors, independently of diet, is highly complex. In Falony et al.’s (2016) study of population-level microbiota variation, dietary intakes were less predictive of gut microbial composition than other intersecting characteristics, such as blood composition (eg. blood cell counts and serum lipid concentrations), stool consistency and pharmaceutical use. While we adjusted for a variety of potential confounders that may exert their own influences on microbial composition, we were likely unable to account for all pertinent factors. Next, detecting diet-microbiota associations is limited by the extent to which particular microbial species are identified and can be isolated. Emerging research has demonstrated that significant diet-microbiota associations may pertain to microbial taxa that have only recently been characterised. Notably, Asnicar et al.’s (2021) Personalised Responses to Dietary Composition Trial (PREDICT 1) used metagenomic analyses to identify diet-microbiota associations, and observed that the strongest correlation in their dietary data was between coffee intake and a previously unexamined species of *Lawsonibacter.* Our use of 16S rRNA gene amplicon sequencing may also have limited our ability to detect diet-microbiota associations. Asnicar et al.’s (2021) metagenomic sequencing identified significant correlations between HEI scores and microbial composition, as well as distinct clustering between food groups and microbial clades.

Lastly, current and previous homelessness in this cohort underscores the uniqueness of U.S. Veterans and the challenges they face. The association between homelessness and poor diet is well-established (Bottino et al., 2019). The impact on the gut microbiome, however, is unknown, and a comprehensive analysis of non-dietary influences on microbiota was outside the scope of this paper. Nevertheless, this is a potentially significant avenue of research that merits further investigation.

A major strength of this study is its focus on military Veterans, an underexamined population in microbiome and nutrition research. While our findings suggest Veterans’ diets largely align with the eating habits of the general population, larger-scale studies are needed to assess if and how Veterans’ shared occupational, environmental and psychological experiences shape their dietary habits and gut microbiota. To the best our knowledge, this is the first study of gut microbiota and diet in this population.

We must also acknowledge this study’s limitations. FFQs assess dietary habits for the previous 12 months, whereas microbiome analyses were conducted on a single stool sample. Repeated analyses of multiple stool samples, as well as longitudinal dietary data, would have been preferable for identifying diet-microbiota associations. Additionally, as our dietary analyses were based on a standardised nutrient database, we were not able to assess the impact of the numerous non-nutritive compounds found in food (eg, flavouring additives and preservatives) that may exert their own influence on microbial composition and diversity (Johnson et al., 2019). Lastly, despite our efforts to mitigate the effects of shipping samples by performing “deblooming” analysis, we were unable to prevent shipping from being a significant predictor of community level composition.

## Conclusion

Overall, the majority of the cohort consumed a diet similar to that of the general U.S. population; this diet was characterised by poor adherence to nutritional guidelines for consumption of fiber-rich foods, sodium and added sugar. We did not observe significant associations between dietary variables and gut microbiota diversity in this study of a cohort of Veterans from the US-VMP. Nevertheless, we observed that the relative abundance of particular phyla was inversely associated with HEI scores, and added sugar intake. Both of these are core factors used to define and characterise populations’ eating habits, and therefore merit further investigation. Lastly, while we did not observe significant diet-microbiota associations, future metagenomic analyses may be able to identify more precise correlations. In particular, more comprehensive analyses are needed that examine relations between dietary data and the microbiome among specific cohorts of Veterans, including those with a history of homelessness.

## Abbreviations

FDR: false discovery rate
FFQ: Food Frequency Questionnaire
HEI: Healthy Eating Index
MaAsLin: multivariate association with linear models
PD: phylogenetic diversity
QIIME: Quantitative Insights into Microbial Ecology
US-VMP: United States-Veteran Microbiome Project

## Research transparency and reproducibility

The Veterans Health Administration has rules about data security that aim to protect the privacy of Veteran health information. Thus, the personal health data used for this study cannot be made publicly available. Please contact the authors for further information or collaboration.

## Supplementary material

For supplementary material accompanying this paper visit https://doi.org/10.1017/gmb.2021.1. click here to view supplementary material

## References

[r1] Afgan E, Baker D, Batut B, Van den Beek M, Bouvier D, Čech M, Chilton J, Clements D, Coraor N, Grüning BA, Guerler A, Hillman-Jackson J, Hiltemann S, Jalili V, Rasche H, Soranzo N, Goecks J, Taylor J, Nekrutenko A and Blankenberget D (2018) The Galaxy platform for accessible, reproducible and collaborative biomedical analyses: 2018 update. Nucleic Acids Research 46(W1), W537–W544. 10.1093/nar/gky379 29790989 PMC6030816

[r2] Amir A, McDonald D, Navas-Molina JA, Debelius J, Morton JT, Hyde E, Robbins-Pianka A and Knight R (2017a) Correcting for microbial blooms in fecal samples during room-temperature shipping. MSystems 2(2), e00199-16. 28289733 10.1128/mSystems.00199-16 PMC5340865

[r3] Amir A, McDonald D, Navas-Molina JA, Kopylova E, Morton JT, Xu ZZ, Kightley EP, Thompson LR, Hyde ER, Gonzalez A and Knight R (2017b) Deblur rapidly resolves single-nucleotide community sequence patterns. MSystems 2(2), e00191-16. 28289731 10.1128/mSystems.00191-16 PMC5340863

[r4] Asnicar F, Berry SE, Valdes AM., Nguyen LH, Piccinno G, Drew DA, Leeming E, Gibson R, Le Roy C, Al Khatib H, Francis L, Mazidi M, Mompeo O, Valles-Colomer M, Tett A, Beghini F, Dubois L, Bazzani D, Thomas AM, Mirzayi C, Khleborodova A, Oh S, Hine R, Bonnett C, Capdevila J, Danzanvilliers S, Giordano F, Geistlinger L, Waldron L, Davies R, Hadjigeorgiou G, Wolf J, Ordovás JM, Gardner C, Franks PW, Chan AT, Huttenhower C, Spector TD and Segata N (2021) Microbiome connections with host metabolism and habitual diet from 1,098 deeply phenotyped individuals. Nature Medicine 27(2), 321–332. 10.1038/s41591-020-01183-8 PMC8353542 33432175

[r5] Belizário JE and Napolitano M (2015) Human microbiomes and their roles in dysbiosis, common diseases, and novel therapeutic approaches. Frontiers in Microbiology 6, 1050. 26500616 10.3389/fmicb.2015.01050 PMC4594012

[r6] Benjamini Y and Hochberg Y (1995) Controlling the false discovery rate: a practical and powerful approach to multiple testing. Journal of the Royal Statistical Society: Series B (Methodological) 57(1), 289–300.

[r7] Bloomfield HE, Kane R, Koeller E, Greer N, MacDonald R and Wilt T (2016) Benefits and harms of the mediterranean diet compared to other diets. VA ESP Project#09-009. 27559560

[r8] Bokulich NA, Maldonado J, Kang D-W, Krajmalnik-Brown R and Caporaso JG (2019) Rapidly processed stool swabs approximate stool microbiota profiles. Msphere 4(2), e00208-19. 30971445 10.1128/mSphere.00208-19 PMC6458435

[r9] Bolyen E, Rideout JR, Dillon MR, Bokulich NA, Abnet CC, Al-Ghalith GA, Alexander H, Alm EJ, Arumugam M, Asnicar F, Bai Y, Bisanz JE, Bittinger K, Brejnrod A, Brislawn CJ, Brown CT, Callahan BJ, Caraballo-Rodríguez AM, Chase J, Cope EK, Da Silva R, Diener C, Dorrestein PC, Douglas GM, Durall DM, Duvallet C, Edwardson CF, Ernst M, Estaki M, Fouquier J, Gauglitz JM, Gibbons SM, Gibson DL, Gonzalez A, Gorlick K, Guo J, Hillmann B, Holmes S, Holste H, Huttenhower C, Huttley GA, Janssen S, Jarmusch AJ, Jiang L, Kaehler BD, Kang KB, Keefe CR, Keim P, Kelley ST, Knights D, Koester I, Kosciolek T, Kreps J, Langille MGI, Lee J, Ley R, Liu Y-X, Loftfield E, Lozupone C, Maher M, Marotz C, Martin BD, McDonald D, McIver LJ, Melnik AV, Metcalf JL, Morgan SC, Morton JT, Naimey AT, Navas-Molina JA, Nothias LF, Orchanian SB, Pearson T, Peoples SL, Petras D, Preuss ML, Pruesse E, Rasmussen LB, Rivers A, Robeson MS, II, Rosenthal P, Segata N, Shaffer M, Shiffer A, Sinha R, Song SJ, Spear JR, Swafford AD, Thompson LR, Torres PJ, Trinh P, Tripathi A, Turnbaugh PJ, Ul-Hasan S, van der Hooft JJJ, Vargas F, Vázquez-Baeza Y, Vogtmann E, von Hippel M, Walters W, Wan Y, Wang M, Warren J, Weber KC, Williamson CHD, Willis AD, Xu ZZ, Zaneveld JR, Zhang Y, Zhu Q, Knight R and Caporaso JG (2019) Reproducible, interactive, scalable and extensible microbiome data science using QIIME 2. Nature Biotechnology 37(8), 852–857. 10.1038/s41587-019-0209-9 PMC7015180 31341288

[r10] Bottino CJ, Fleegler EW, Cox JE and Rhodes ET (2019) The relationship between housing instability and poor diet quality among urban families. Academic Pediatrics 19(8), 891–898. 30986548 10.1016/j.acap.2019.04.004

[r11] Brenner LA, Hoisington AJ, Stearns-Yoder KA, Stamper CE, Heinze JD, Postolache TT, Hadidi DA, Hoffmire CA, Stanislawski MA and Lowry CA (2018) Military-related exposures, social determinants of health, and dysbiosis: The United States-veteran microbiome project (US-VMP). Frontiers in Cellular and Infection Microbiology 8, 400. 30510919 10.3389/fcimb.2018.00400 PMC6252388

[r13] Center for Disease Control and Prevention (2016) National Health and Nutrition Examination Survey Data 2015–2016. Hyattsville, MD: U.S. Department of Health and Human Services.

[r14] Claesson MJ, Jeffery IB, Conde S, Power SE, O’Connor EM, Cusack S, Harris HMB, Coakley M, Lakshminarayanan B, O’Sullivan O, Fitzgerald GF, Deane J, O’Connor M, Harnedy N, O’Connor K, O’Mahony D, van Sinderen D, Wallace M, Brennan L, Stanton C, Marchesi JR, Fitzgerald AP, Shanahan F, Hill C, Ross RP and O’Toole PW (2012) Gut microbiota composition correlates with diet and health in the elderly. Nature 488(7410), 178–184. 10.1038/nature11319 22797518

[r15] Collins RA, Baker B, Coyle DH, Rollo ME and Burrows TL (2020) Dietary assessment methods in military and veteran populations: a scoping review. Nutrients 12(3), 769. 32183380 10.3390/nu12030769 PMC7146105

[r16] Davis SC, Yadav JS, Barrow SD and Robertson BK (2017) Gut microbiome diversity influenced more by the westernized dietary regime than the body mass index as assessed using effect size statistic. Microbiology, 6(4), e00476. 10.1002/mbo3.476 PMC5552927 28677210

[r17] Dinan TG and Cryan JF (2017) The microbiome-gut-brain axis in health and disease. Gastroenterology Clinics 46(1), 77–89. 28164854 10.1016/j.gtc.2016.09.007

[r18] Egshatyan L, Kashtanova D, Popenko A, Tkacheva O, Tyakht A, Alexeev D, Karamnova N, Kostryukova E, Babenko V, Vakhitova M and Boytsov S (2016) Gut microbiota and diet in patients with different glucose tolerance. Endocrine Connections 5(1), 1–9. 26555712 10.1530/EC-15-0094 PMC4674628

[r19] Faith JJ, Guruge JL, Charbonneau M, Subramanian S, Seedorf H, Goodman AL., Clemente JC, Knight R, Heath AC, Leibel RL., Rosenbaum M and Gordon JI (2013) The long-term stability of the human gut microbiota. Science 341(6141), 1237439. 23828941 10.1126/science.1237439 PMC3791589

[r20] Falony G, Joossens M, Vieira-Silva S, Wang J, Darzi Y, Faust K, Kurilshikov A, Bonder MJ, Valles-Colomer M, Vandeputte D, Tito RY, Chaffron S, Rymenans L, Verspecht C, De Sutter L, Lima-Mendez G, D’hoe K, Jonckheere K, Homola D, Garcia R, Tigchelaar EF, Eeckhaudt L, Fu J, Henckaerts L, Zhernakova A, Wijmenga C and Raes J (2016) Population-level analysis of gut microbiome variation. Science 352(6285), 560–564. 27126039 10.1126/science.aad3503

[r21] Gebhardt S, Lemar L, Haytowitz P, Pehrsson P, Nickle M, Showell B, Thomas R, Exler J and Holden J (2008) *USDA National Nutrient Database for Standard Reference, Release 21.* United States Department of Agriculture Agricultural Research Service.

[r22] Graf D, Di Cagno R, Fåk F, Flint HJ, Nyman M, Saarela M and Watzl B (2015) Contribution of diet to the composition of the human gut microbiota. Microbial Ecology in Health and Disease 26(1), 26164. 25656825 10.3402/mehd.v26.26164 PMC4318938

[r23] Janssen S, McDonald D, Gonzalez A, Navas-Molina JA, Jiang L, Xu ZZ, Winker K, Kado DM, Manary M, Mirarab S and Knight R (2018). Phylogenetic placement of exact amplicon sequences improves associations with clinical information. mSystems 3, e00021-18. 29719869 10.1128/mSystems.00021-18 PMC5904434

[r24] Johnson AJ, Vangay P, Al-Ghalith GA, Hillmann BM, Ward TL, Shields-Cutler RR, Kim AD, Shmagel AK and Syed AN (2019) Daily sampling reveals personalized diet-microbiome associations in humans. Cell Host & Microbe 25(6), 789–802. 31194939 10.1016/j.chom.2019.05.005

[r25] Krebs-Smith SM, Pannucci TE, Subar AF, Kirkpatrick SI, Lerman JL, Tooze JA, Wilson MM and Reedy J (2018) Update of the healthy eating index: HEI-2015. Journal of the Academy of Nutrition and Dietetics 118(9), 1591–1602. 30146071 10.1016/j.jand.2018.05.021 PMC6719291

[r26] Mandal S, Van Treuren W, White RA, Eggesbø M, Knight R and Peddada S (2015) Analysis of composition of microbiomes: a novel method for studying microbial composition. Microbial Ecology in Health and Disease 26(1), 27663. 26028277 10.3402/mehd.v26.27663 PMC4450248

[r27] Mazidi M, Rezaie P, Kengne AP, Mobarhan MG and Ferns GA (2016) Gut microbiome and metabolic syndrome. Diabetes & Metabolic Syndrome: Clinical Research & Reviews 10(2), S150–S157. 10.1016/j.dsx.2016.01.024 26916014

[r28] Menni C, Louca P, Berry SE, Vijay A, Astbury S, Leeming ER, Gibson R, Ascinar F, Piccinno G, Wolf J, Davies R, Mangino M, Segata N, Spector TD and Valdes AM (2021) High intake of vegetables is linked to lower white blood cell profile and the effect is mediated by the gut microbiome. BMC Medicine 19(1), 1–10. 33568158 10.1186/s12916-021-01913-w PMC7875684

[r29] Oksanen J, Blanchet FG., Friendly M, Kindt R, Legendre P, McGlinn D, Minchin PR, O’Hara RB, Simpson GL, Solymos P and Stevens MHH (2018). vegan: Community Ecology Package. R package version 2.5-2.

[r30] Partula V, Mondot S, Torres MJ, Kesse-Guyot E, Deschasaux M, Assmann K, Latino-Martel P, Buscail C, Julia C, Galan P, Hercberg S, Rouilly V, Thomas S, Quintana-Murci L, Albert ML, Duffy D, Lantz O and Touvier M (2019) Associations between usual diet and gut microbiota composition: Results from the milieu Intérieur cross-sectional study. The American Journal of Clinical Nutrition 109(5), 1472–1483. 31051503 10.1093/ajcn/nqz029

[r31] Quast C, Pruesse E, Yilmaz P, Gerken J, Schweer T, Yarza P, Peplies J and Glöckner FO (2012) The SILVA ribosomal RNA gene database project: improved data processing and web-based tools. Nucleic Acids Research 41(D1), D590–D596. 23193283 10.1093/nar/gks1219 PMC3531112

[r32] Rinninella E, Raoul P, Cintoni M, Franceschi F, Miggiano GAD, Gasbarrini A and Mele MC (2019) What is the healthy gut microbiota composition? A changing ecosystem across age, environment, diet, and diseases. Microorganisms 7(1), 14. 30634578 10.3390/microorganisms7010014 PMC6351938

[r33] Singh RK, Chang HW, Yan D, Lee KM, Ucmak D, Wong K, Abrouk M, Farahnik B, Nakamura M, Zhu TH, Bhutani T and Liao W (2017) Influence of diet on the gut microbiome and implications for human health. Journal of Translational Medicine 15(1), 73. 10.1186/s12967-017-1175-y 28388917 PMC5385025

[r34] Thaiss CA, Zmora N, Levy M and Elinav E (2016) The microbiome and innate immunity. Nature 535(7610), 65–74. 27383981 10.1038/nature18847

[r35] US Department of Agriculture (2015) 2015–2020 Dietary Guidelines for Americans, 8th Edn. Washington, DC: US Department of Agriculture. Available at http://www.health.gov/DietaryGuidelines

[r36] Veteran Population – National Center for Veterans Analysis and Statistics (2016) Available at http://www.va.gov/vetdata/veteran_population.asp

[r37] VIReC (2014 (Rev. September 2017). Calculating a Comorbidity Index for Risk Adjustment Using VA or Medicare Data. Hines, IL: VIReC.

[r38] Vujkovic-Cvijin I, Sklar J, Jiang L, Natarajan L, Knight R and Belkaid Y (2020) Host variables confound gut microbiota studies of human disease. Nature 587(7834), 448–454. 33149306 10.1038/s41586-020-2881-9 PMC7677204

[r39] Willett WC, Sampson L, Stampfer MJ, Rosner B, Bain C, Witschi J, Hennekens CH and Speizer FE (1985) Reproducibility and validity of a semiquantitative food frequency questionnaire. American Journal of Epidemiology 122(1), 51–65. 4014201 10.1093/oxfordjournals.aje.a114086

[r40] Wu GD, Chen J, Hoffmann C, Bittinger K, Chen Y-Y, Keilbaugh SA, Bewtra M, Knights D, Walters WA, Knight R, Sinha R, Gilroy E, Gupta K, Baldassano R, Nessel L, Li H, Bushman FD and Lewis JD (2011) Linking long-term dietary patterns with gut microbial enterotypes. Science 334(6052), 105–108. 10.1126/science.1208344 21885731 PMC3368382

