# Supplementary material for: Dietary habits and the gut microbiota in military Veterans: results from the United States-Veteran Microbiome Project (US-VMP)
Source: Gut Microbiome (Camb). 2021 Apr 28;2:e1. doi: 10.1017/gmb.2021.1 (PMC11406408; doi:10.1017/gmb.2021.1)
Supplement: Supplementary file 1 [file gmbsup.zip › S2632289721000013sup002.docx]

**Supplemental Table 1.** **Table of correlation results of sOTUs belonging to the family of Enterobacteriaceae against transit days.**

**Supplemental Table 2.** **Classification of Food Frequency Questionnaire items, based on nutritional characteristics.**

| **Fruit** | Raisins / grapes | **Animal foods** | Meat & seafood / animal protein group |
| --- | --- | --- | --- |
|  | Prunes |  | Processed meats group |
|  | Bananas |  | Fermented dairy group |
|  | Cantaloupe / other melons |  | Milk (skim, 2%, whole) |
|  | Avocado |  | Cream |
|  | Apples / pears / apple sauce | **Plant proteins** | Tofu / soybeans / soy-based ‘meats’ |
|  | Oranges |  | Soymilk |
|  | Grapefruit |  | Beans / peas |
|  | Strawberries |  | Peanuts / peanut butter |
|  | Blueberries |  | Walnuts / other nuts |
|  | Peaches / plums | **Complex carbohydrates** | Oatmeal / cooked oat bran |
|  | Apricots |  | Other cooked cereal |
| **Non-starchy vegetables** | Tomatoes |  | Rye / pumpernickel bread |
|  | Tomato juice / sauce / salsa |  | Whole wheat/whole grain bread |
|  | Broccoli |  | Brown rice |
|  | Cabbage / coleslaw |  | Other cooked whole grain |
|  | Cauliflower |  | Corn |
|  | Brussels sprouts |  | Popcorn |
|  | Carrots |  | Bran / wheat germ |
|  | Mixed vegetables |  | Potatoes (excluding French fries & chips) |
|  | Orange/winter squash |  | Sweet potatoes |
|  | Eggplant / zucchini / summer squash | **Fried foods** | French fried potatoes |
|  | Spinach |  | Potato chips |
|  | Kale / mustard greens / chard |  | Breaded & fried meats/seafood |
|  | Iceberg lettuce / head lettuce | **Sweets & desserts** | Chocolate (milk & dark) |
|  | Romaine / leaf lettuce |  | Chocolate-based candy bars |
|  | Celery |  | Candy without chocolate |
|  | Peppers |  | Jams/jellies/fruit preserves |
|  | Onions |  | Cookies (commercial & homemade) |
| **Fermented dairy** | Plain yogurt |  | Brownies |
|  | Flavored yogurt |  | Doughnuts |
|  | Cottage cheese / soft cheeses |  | Cake (commercial & homemade) |
|  | Hard cheeses |  | Pie |
| **Meat & seafood /**  **Animal protein** | Chicken |  | Sweet rolls / coffee cake / other pastry |
|  | Chicken / turkey |  | Breakfast cereal bars |
|  | Beef / lamb |  | Pancakes / waffles |
|  | Pork |  | Frozen yogurt |
|  | Liver (poultry, beef, pork) |  | Ice cream |
|  | Tuna | **Sugar-sweetened /**  **sugary beverages** | Cola-type soft drinks |
|  | Dark meat fish |  | Other soft drinks |
|  | Other fish |  | Fruit punch |
|  | Shrimp / lobster / scallops / clams |  | Fruit juice |
|  | Eggs | **Alcoholic beverages** |  |
| **Processed meats** | Bacon |  | Beer (regular & light) |
|  | Hot dogs |  | Red wine |
|  | Salami / bologna / processed deli meat |  | White wine |
|  | Sausage |  | Liquor |
|  | Fish sticks |  |  |

**Supplemental Table 3. Demographic and medical characteristics of the United States-Veteran Microbiome Project (US-VMP) cohort.**

| **Variables** | **Sample (*N* = 330)** | |
| --- | --- | --- |
| **Age (mean years ± SD)** | 47.6 | ± 13.4 |
| **Male sex** | 274 | (83.0%) |
| **Racial background** |  | |
| Caucasian/White | 217 | (65.8%) |
| Black/African American | 62 | (18.8%) |
| Native American/Alaskan Native | 6 | (1.8%) |
| Asian | 4 | (1.2%) |
| Multiracial/Other | 41 | (12.4%) |
| **Marital status** | **(*N* = 329)** | |
| Single | 82 | (24.9%) |
| Married/Cohabitating | 144 | (43.8%) |
| Widowed | 15 | (4.6%) |
| Divorced/Separated | 88 | (26.8%) |
| **Education** | **(*N* = 330)** | |
| ≤ High school diploma | 41 | (12.4%) |
| Some college, no degree | 121 | (36.7%) |
| Associate’s degree | 52 | (15.8%) |
| Bachelor’s degree | 78 | (23.6%) |
| ≥ Master’s degree | 38 | (11.5%) |
| **Employment status** | **(*N* = 329)** | |
| Full-time | 79 | (24.0%) |
| Part-time | 34 | (10.3%) |
| Unemployed, not currently seeking | 74 | (22.5%) |
| Unemployed, seeking employment | 53 | (16.1%) |
| Retired | 89 | (27.1%) |
| **Homelessness** | **(*N* = 330)** | |
| Currently homeless | 29 | (8.8%) |
| Ever previously homeless | 138 | (41.8%) |
| **Military service era** | **(*N* = 330)** | |
| Pre-Vietnam | 6 | (1.8%) |
| Vietnam | 45 | (13.6%) |
| Post-Vietnam/Pre-Gulf War | 127 | (38.5%) |
| First Gulf War | 116 | (35.2%) |
| Post-9/11 | 130 | (39.4%) |
| **Number of military deployments (mean ± SD)** | 2.0 | ± 2.9 |
| **Anthropometrics** | **(*N* = 322)** | |
| Body Mass Index (BMI) | 28.9 | ± 6.0 |
| **Smoking Status** | **(*N* = 330)** | |
| Current/Former | 262 | (79.4%) |
| Never | 62 | (18.8%) |
| Unknown | 6 | (1.8%) |
| **Medical Comorbidities** | **(*N* = 322)** | |
| Comorbidity Index^*^ | 1.0 | ± 1.7 |

^*^ Based on the Charlson Comorbidity Index (CCI), a risk adjustment measure for predicting mortality associated with 19 different medical conditions

**Supplemental Table 4. Associations^*^ between dietary variables^†^ and α-diversity indices in the United States-Veteran Microbiome Project (US-VMP) cohort (*N* = 330)**

| **Dietary Variable^1^** | **Observed Species** | | | | | **Shannon Diversity** | | | | | **Faith’s Phylogenetic Diversity** | | | | |
| --- | --- | --- | --- | --- | --- | --- | --- | --- | --- | --- | --- | --- | --- | --- | --- |
|  | ***R^2^*** | **Effect Size** | **SE** | ***p*** | **FDR-*p***^§^ | ***R^2^*** | **Effect Size** | **SE** | ***p*** | **FDR-*p***^§^ | ***R^2^*** | **Effect Size** | **SE** | ***p*** | **FDR-*p***^§^ |
| **Healthy Eating Index** | 0.053 | 0.18 | 0.19 | 0.344 | 0.726 | 0.042 | 0.003 | 0.003 | 0.367 | 0.789 | 0.045 | 0.04 | 0.05 | 0.402 | 0.735 |
| **Adherence to a Western dietary pattern**^‡^ |  |  |  |  |  |  |  |  |  |  |  |  |  |  |  |
| Low | 0.057 | -3.79 | 7.09 | 0.593 | 0.771 | 0.040 | 0.03 | 0.13 | 0.840 | 0.970 | 0.046 | 0.01 | 1.91 | 0.995 | 0.995 |
| Moderate | 0.057 | -5.90 | 7.74 | 0.447 | 0.726 | 0.040 | -0.08 | 0.14 | 0.587 | 0.857 | 0.046 | -0.28 | 2.09 | 0.895 | 0.995 |
| High | 0.057 | 3.29 | 6.73 | 0.626 | 0.775 | 0.040 | -0.004 | 0.13 | 0.970 | 0.970 | 0.046 | 1.43 | 1.82 | 0.432 | 0.735 |
| **Food and nutrient intakes** |  |  |  |  |  |  |  |  |  |  |  |  |  |  |  |
| Added sugars (g) | 0.067 | -0.13 | 0.05 | 0.019 | 0.494 | 0.060 | -0.003 | 0.001 | 0.011 | 0.143 | 0.059 | -0.033 | 0.01 | 0.023 | 0.549 |
| Animal fat (g) | 0.059 | 0.50 | 0.19 | 0.096 | 0.511 | 0.053 | 0.01 | 0.003 | 0.033 | 0.286 | 0.051 | 0.09 | 0.05 | 0.093 | 0.549 |
| Caffeine (mg) | 0.056 | 0.02 | 0.01 | 0.150 | 0.557 | 0.060 | 0.001 | 0.0003 | 0.010 | 0.143 | 0.047 | 0.004 | 0.003 | 0.262 | 0.681 |
| Total flavonoids (mg) | 0.053 | 0.01 | 0.01 | 0.344 | 0.726 | 0.040 | 0.0001 | 0.0001 | 0.661 | 0.905 | 0.044 | 0.002 | 0.002 | 0.529 | 0.764 |
| Fiber from grains (g) | 0.053 | 0.69 | 0.76 | 0.366 | 0.726 | 0.040 | 0.009 | 0.01 | 0.539 | 0.857 | 0.044 | 0.16 | 0.21 | 0.452 | 0.735 |
| Fiber from fruit, vegetables, and legumes (g) | 0.050 | 0.03 | 0.28 | 0.910 | 0.910 | 0.039 | -0.0003 | 0.01 | 0.953 | 0.970 | 0.043 | -0.01 | 0.07 | 0.939 | 0.995 |
| Plant-based fat (g) | 0.058 | -0.23 | 0.14 | 0.100 | 0.511 | 0.045 | -0.003 | 0.003 | 0.189 | 0.754 | 0.050 | -0.06 | 0.04 | 0.129 | 0.549 |
| Sodium (mg) | 0.050 | -0.001 | 0.005 | 0.836 | 0.869 | 0.040 | 0.0001 | 0.00003 | 0.564 | 0.857 | 0.043 | -0.00005 | 0.001 | 0.971 | 0.995 |
| Alcoholic beverages (servings) | 0.051 | 0.71 | 1.28 | 0.577 | 0.771 | 0.039 | 0.004 | 0.02 | 0.855 | 0.970 | 0.043 | 0.16 | 0.34 | 0.646 | 0.849 |
| Artificially sweetened beverages (servings) | 0.061 | -5.73 | 3.04 | 0.061 | 0.511 | 0.043 | -0.07 | 0.06 | 0.247 | 0.754 | 0.052 | -1.45 | 0.82 | 0.078 | 0.549 |
| Complex carbohydrates (servings) | 0.052 | 1.25 | 1.78 | 0.485 | 0.742 | 0.041 | 0.03 | 0.03 | 0.425 | 0.789 | 0.044 | 0.36 | 0.48 | 0.449 | 0.735 |
| Fermented dairy (servings) | 0.053 | 4.17 | 3.94 | 0.291 | 0.726 | 0.043 | 0.08 | 0.07 | 0.290 | 0.754 | 0.048 | 1.39 | 1.06 | 0.190 | 0.549 |
| Fruit (servings) | 0.053 | 1.88 | 1.86 | 0.315 | 0.726 | 0.040 | 0.02 | 0.03 | 0.593 | 0.857 | 0.046 | 0.50 | 0.50 | 0.149 | 0.549 |
| Non-starchy vegetables (servings) | 0.050 | 0.31 | 1.34 | 0.814 | 0.869 | 0.039 | -0.002 | 0.02 | 0.938 | 0.970 | 0.043 | 0.09 | 0.36 | 0.801 | 0.947 |
| Plant protein (servings) | 0.055 | -2.72 | 2.26 | 0.230 | 0.726 | 0.042 | -0.04 | 0.04 | 0.320 | 0.756 | 0.048 | -0.82 | 0.61 | 0.177 | 0.549 |
| Fruit and vegetables (servings) | 0.051 | 0.58 | 0.90 | 0.519 | 0.750 | 0.039 | 0.003 | 0.02 | 0.836 | 0.970 | 0.044 | 0.16 | 0.24 | 0.515 | 0.764 |
| Fried foods (servings) | 0.058 | -9.68 | 6.17 | 0.118 | 0.511 | 0.043 | -0.13 | 0.11 | 0.263 | 0.754 | 0.050 | -2.40 | 1.66 | 0.149 | 0.549 |
| Meat and seafood (servings) | 0.050 | 0.51 | 1.97 | 0.794 | 0.869 | 0.041 | 0.03 | 0.04 | 0.410 | 0.789 | 0.043 | 0.24 | 0.53 | 0.653 | 0.849 |
| Processed meats (servings) | 0.050 | -1.30 | 5.20 | 0.803 | 0.869 | 0.039 | -0.01 | 0.10 | 0.890 | 0.970 | 0.043 | -0.41 | 1.40 | 0.772 | 0.947 |
| Animal foods (servings) | 0.052 | 1.08 | 1.34 | 0.422 | 0.726 | 0.044 | 0.03 | 0.02 | 0.208 | 0.754 | 0.045 | 0.32 | 0.36 | 0.380 | 0.735 |
| Sugar-sweetened/sugary beverages (servings) | 0.052 | -1.41 | 1.85 | 0.447 | 0.726 | 0.052 | -0.07 | 0.03 | 0.045 | 0.293 | 0.045 | -0.40 | 0.50 | 0.427 | 0.735 |
| Sweets and desserts (servings) | 0.059 | -2.56 | 1.53 | 0.096 | 0.511 | 0.046 | -0.04 | 0.03 | 0.152 | 0.754 | 0.050 | -0.65 | 0.41 | 0.117 | 0.549 |

^*^ Linear regression models controlled for total energy intake, demographic variables, medical comorbidities, and number of military deployments

^†^ All dietary variables analyzed as average daily intakes

^‡^ Reference category: Very Low adherence to a Western dietary pattern

^§^ *P* value after multivariate adjustment and correction for multiple comparison testing using a False Discovery Rate (FDR) of 0.10

Abbreviations: g – grams, mg - milligrams

**Supplemental Table 5. Associations* between dietary variables^†^ and β-diversity indices in the United States-Veteran Microbiome Project (US-VMP) cohort (*N* = 330).**

| **Dietary Variable^†^** | **Unweighted UniFrac** | | | **Weighted UniFrac** | | |
| --- | --- | --- | --- | --- | --- | --- |
|  | ***R^2^*** | ***p*** | **FDR-*p***^‡^ | ***R^2^*** | ***p*** | **FDR-*p***^‡^ |
| **Healthy Eating Index** | 0.002 | 0.715 | 0.97 | 0.01 | 0.208 | 0.631 |
| **Adherence to a Western dietary pattern** | 0.008 | 0.314 | 0.97 | 0.008 | 0.28 | 0.631 |
| **Food and nutrient intakes** |  |  |  |  |  |  |
| Added sugars (g) | 0.005 | 0.46 | 0.97 | 0.0003 | 0.955 | 0.984 |
| Animal fat (g) | 0.002 | 0.769 | 0.97 | 0.005 | 0.473 | 0.631 |
| Caffeine (mg) | 0.010 | 0.173 | 0.97 | 0.005 | 0.455 | 0.631 |
| Total flavonoids (mg) | 0.006 | 0.402 | 0.97 | 0.006 | 0.395 | 0.631 |
| Fiber from grains (g) | 0.001 | 0.918 | 0.97 | 0.002 | 0.733 | 0.838 |
| Fiber from fruit, vegetables, and legumes (g) | 0.0003 | 0.962 | 0.97 | 0.016 | 0.074 | 0.296 |
| Plant-based fat (g) | 0.002 | 0.749 | 0.97 | 0.003 | 0.657 | 0.788 |
| Sodium (mg) | <0.0001 | 0.993 | 0.97 | 0.001 | 0.873 | 0.952 |
| Alcoholic beverages (servings) | 0.005 | 0.419 | 0.97 | 0.007 | 0.328 | 0.631 |
| Artificially sweetened beverages (servings) | 0.011 | 0.172 | 0.97 | 0.016 | 0.069 | 0.296 |
| Complex carbohydrates (servings) | 0.002 | 0.75 | 0.97 | 0.006 | 0.364 | 0.631 |
| Fermented dairy (servings) | 0.009 | 0.212 | 0.97 | 0.010 | 0.214 | 0.631 |
| Fruit (servings) | 0.001 | 0.893 | 0.97 | 0.005 | 0.437 | 0.631 |
| Non-starchy vegetables (servings) | 0.0004 | 0.94 | 0.97 | 0.021 | 0.034 | 0.296 |
| Plant protein (servings) | 0.003 | 0.587 | 0.97 | 0.008 | 0.29 | 0.631 |
| Fruit and vegetables (servings) | 0.001 | 0.924 | 0.97 | 0.017 | 0.064 | 0.296 |
| Fried foods (servings) | 0.004 | 0.501 | 0.97 | 0.003 | 0.653 | 0.788 |
| Meat and seafood (servings) | 0.001 | 0.855 | 0.97 | 0.017 | 0.073 | 0.296 |
| Processed meats (servings) | 0.004 | 0.516 | 0.97 | 0.019 | 0.049 | 0.296 |
| Animal foods (servings) | 0.0003 | 0.946 | 0.97 | 0.005 | 0.473 | 0.631 |
| Sugar-sweetened/sugary beverages (servings) | 0.001 | 0.861 | 0.97 | 0.0001 | 0.984 | 0.984 |
| Sweets and desserts (servings) | 0.003 | 0.654 | 0.97 | 0.005 | 0.407 | 0.631 |

^*^ Models controlled for total energy intake, demographic variables, medical comorbidities, and number of military deployments

^†^ All dietary variables analyzed as average daily intakes

^‡^ *P* value after multivariate adjustment and correction for multiple comparison testing using a False Discovery Rate (FDR) of 0.10

Abbreviations: g – grams, mg - milligrams
