# Supplementary material for: Dietary habits and the gut microbiota in military Veterans: results from the United States-Veteran Microbiome Project (US-VMP)
Source: Gut Microbiome (Camb). 2021 Apr 28;2:e1. doi: 10.1017/gmb.2021.1 (PMC11406408; doi:10.1017/gmb.2021.1)
Supplement: Supplementary file 1 [file gmbsup.zip › S2632289721000013sup001.docx]

**Supplement 1. Bloom analysis for the US-Veteran Microbiome Project (US-VMP)**

Our sampling procedures allowed participants to provide samples in one of two ways: 1) during their in-person visit; or 2) using a provided sampling kit at home and subsequently shipping the sample to the research facility. Although the participants were provided with an ice pack and instructed to ship the sample to the research facility with the ice pack, this protocol was not always followed. Therefore, samples shipped to the research facility had the potential to be above freezing temperature for extended periods of time (1 - 44 days). In contrast, samples collected by participants that elected to provide their sample during the in-person visit were immediately frozen and stored at –80 °C.

Amir et al. (Amir, McDonald, Navas-Molina, Debelius, et al., 2017), reported on the phenomena of “blooming”, where certain taxa that are not obligate anerobic bacteria can bloom in fecal samples if not quickly refrigerated or frozen. Their publication provided detailed explanations, files, and code on how taxa determined to have artificially “bloomed” were removed from the American Gut Project data set. We performed the deblooming analysis as previously described by Amir and colleagues (Amir, McDonald, Navas-Molina, Debelius, et al., 2017). A total of *N* = 95 samples were provided during in-person visits, whereas a total of *N* = 235 samples were shipped. *R^2^* values from canonical correspondence analysis (CCA) of the weighted UniFrac distance matrix were used as a means to objectively determine how deblooming analysis impacted measures of bacterial community structure. We examined *R^2^* values of both the dichotomous variable of shipped (yes (*N* = 235) or no (*N* = 95)) and the continuous variable of transit days (number of days between when the sample was collected by the participant and when the sample was placed into the –80 °C freezer at the research facility; mean ± SD (2.94 ± 3.82 days)). We performed CCA analysis on the data before deblooming analysis, after deblooming analysis of all samples (*N* = 330), and after deblooming analysis of only samples shipped back to the research facility (*N* = 235). The results of this analysis are shown in **Supplemental Figure 1**.

**Supplemental Figure 1.** **Bar plots of *R^2^* values from canonical correspondence analysis (CCA) of the weighted UniFrac distance matrix for the shipped variable. CCA was performed on the data before deblooming analysis, after deblooming analysis on all samples, and after deblooming analysis on only samples shipped to the research facility.**


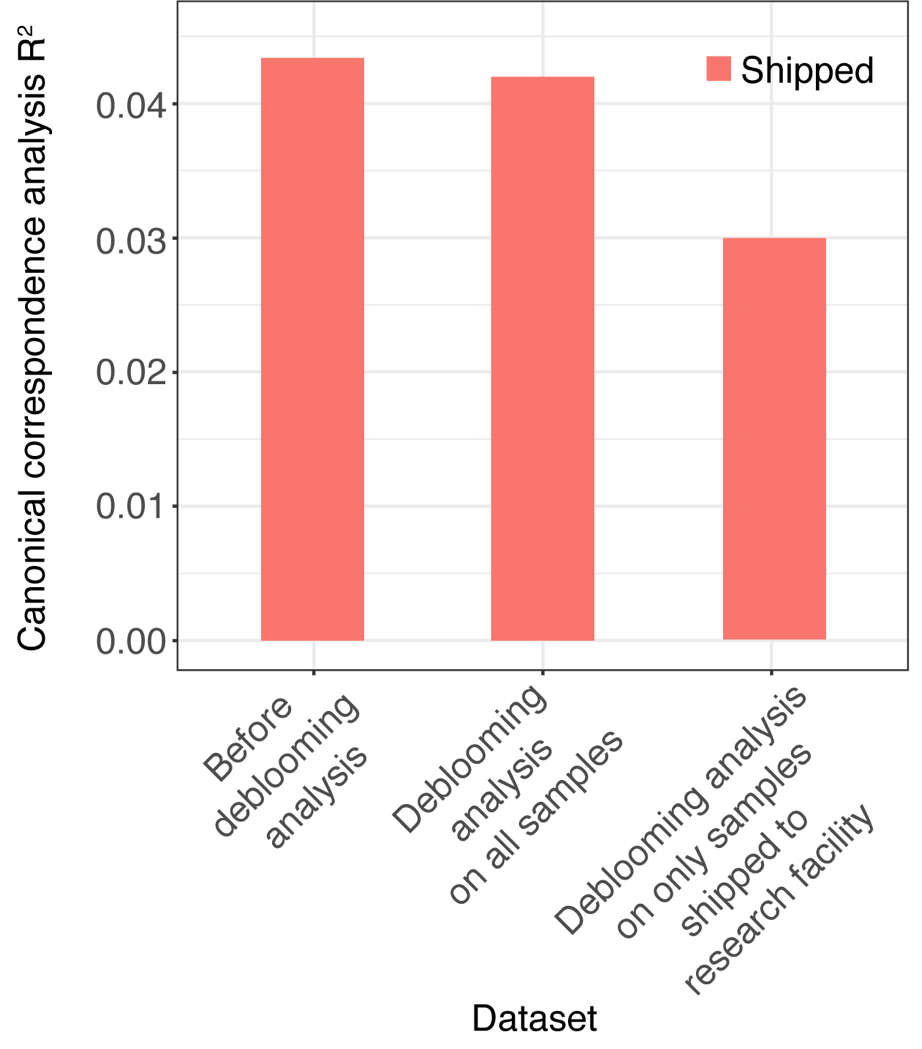


The *R^2^* values from the CCA analysis, which are measures of the variance explained by the factor “shipping” on the bacterial community composition, were reduced by performing the deblooming analysis on only samples shipped back to the research facility. The variance explained (*R^2^* value) was reduced from 4.5% to 3% by performing the deblooming analysis on only samples shipped back to the research facility. Although not ideal, we believe that this approach provided an objective analysis for performing the deblooming analysis in only samples shipped back to the research facility.

Additionally, Bokulich et al. (Bokulich, Maldonado, Kang, Krajmalnik-Brown, & Caporaso, 2019) showed that the taxa that artificially bloom in fecal samples exposed to room temperature conditions for extended periods of time are overrepresented by members of the Enterobacteriaceae family. For this reason, we also examined all the sOTUs in our dataset belonging to the Enterobacteriaceae family for significant correlation with transit days to ensure that our initial deblooming analysis did not overlook other taxa that may have bloomed. We found 21 sOTUs in our dataset belonging to the Enterobacteriaceae family. The results for the correlations run for each sOTU against transit days can be found in **Supplemental Table 1**.

**Supplemental Table 1.** **Table of correlation results of sOTUs belonging to the family of Enterobacteriaceae against transit days.**

There was only 1 sOTU from the Enterobacteriaceae family that was significantly correlated with transit days (sOTU: 4c8288bfbd76958c0c094d87b97650f8; Genus: *Escherichia-Shigella*; Species: Unknown). This sOTU was also identified by Amir et al. (Amir, McDonald, Navas-Molina, Kopylova, et al., 2017), and therefore was already removed from the dataset during the initial deblooming analysis. Consequently, in this report we have used data derived from deblooming of only the samples shipped back to the research facility.
